# Time for Routine *Helicobacter pylori* Screening in Coronary Artery Disease?

**DOI:** 10.1161/CIRCULATIONAHA.123.064944

**Published:** 2023-06-05

**Authors:** Robin Hofmann, Magnus Bäck

**Affiliations:** 1 Department of Clinical Science and Education, Division of Cardiology, Karolinska Institutet, Södersjukhuset, Stockholm, Sweden (R.H.).; 2 Department of Cardiology, Karolinska University Hospital, Stockholm, Sweden (M.B.).

**Keywords:** coronary artery disease, Helicobacter pylori, hemorrhage

Antiplatelet therapy in coronary artery disease involves a tradeoff between the risk of recurrent cardiovascular events and spontaneous bleeding, commonly from the upper gastrointestinal tract. Gastrointestinal injury diagnosed by capsule endoscopy occurs in 94.3% of patients on single antiplatelet therapy during the first year after percutaneous coronary intervention, rising to 99.2% in those on dual antiplatelet therapy,^1^ illustrating the extent of the clinical dilemma. Severe upper gastrointestinal bleeding (UGIB) requiring hospitalization is relatively common, with a 1.5% cumulative incidence in the year after myocardial infarction (MI), and associated with a substantially increased risk of death (hazard ratio, 2.86) and recurrent cardiovascular events (hazard ratio for MI or stroke, 1.17 and 1.80, respectively).^2^ Recommended bleeding avoidance strategies encompass prophylactic use of proton pump inhibitors as well as individualization of dual antiplatelet therapy in terms of shorter duration, de-escalation to less potent therapies, and single treatment with either aspirin alone or aspirin-free strategies.^2,3^

## *H pylori* as a Modifiable Risk Factor to Prevent Upper GI Bleeding After MI

Risk factors for UGIB during antiplatelet treatment include high age, female sex, kidney disease, anemia, and chronic bacterial infection caused by *Helicobacter pylori*.^2,4^ Concomitant *H pylori* infection increases the risk of upper GI bleeding, from 1.8-fold with low-dose aspirin up to 7.4-fold with dual antiplatelet therapy.^4^ In addition to endoscopic sampling, *H pylori* infection can be detected noninvasively with similar high accuracy using fecal *H pylori* antigen test, serology, or urea breath test (sensitivity/specificity ≈90%),^3^ allowing for broad screening.^4^ *H pylori* eradication treatment is recommended using a combination of different antibiotics, depending on individual and regional resistance profiles, and proton pump inhibitors, aiming for successful eradication rates >90% with first-line therapy, and thereby effectively healing ulcers.^4^ Successful *H pylori* eradication may reduce the risk of UGIB complications and allow uninterrupted antiplatelet therapy, both potentially contributing to beneficial effects on subsequent cardiovascular outcomes.^2,4^ In addition, *H pylori* infection may promote development of atherothrombosis through (1) chronic inflammation and direct injury of the vascular wall leading to progression or rupture of atherosclerotic plaque and (2) a systemic inflammatory response in reaction to colonization of the gastric mucosa. These inflammatory processes can induce prothrombotic changes in the blood both related to plasma (ie, hyperfibrinogenemia, altered blood coagulation) and platelet-dependent (ie, induced platelet count, activation, and aggregation [eg, by lipid peroxidation]) homeostasis (summarized in reference^3^), thus contributing to the development of acute coronary syndrome.

Because of a lack of data from randomized clinical trials, *H pylori* screening and eradication is recommended by expert opinion in gastroenterology guidelines, but hitherto largely disregarded in cardiology guidelines.^4^

## Routine *H pylori* Screening and Eradication May Reduce Peptic Ulcer Bleeding, but Questions Remain

*H pylori* screening results recently were presented from the randomized, double-blind, placebo-controlled HEAT (Helicobacter Eradication Aspirin Trial), conducted at 1208 primary care facilities in the United Kingdom. The study reported an active *H pylori* infection detected by urea breath test in 17.8% of 30 166 unselected individuals receiving ≤325 mg daily aspirin.^5^ Together with the previously reported 20% *H pylori* prevalence in contemporary patients with myocardial infarction in Sweden,^4^ these findings support *H pylori* as a widespread risk factor for bleeding complications from cardiovascular prevention measures. The HEAT investigators demonstrate for the first time a significant reduction in UGIB by routine *H pylori* screening and eradication. However, several limitations merit consideration. First, the trial was terminated early because of a lower-than-expected event rate and thus was underpowered for the primary end point. Second, the analyses may not sufficiently account for competing risks of death attributable to conditions other than bleeding. Third, because of the pragmatic design of the trial, the use of gastroprotective therapies to prevent gastric complications was uncontrolled, which may have biased the results. Fourth, the observed treatment effect of a reduction in UGIB was only transient, and the benefit was lost beyond 2.5 years of follow-up.^5^ In particular, the loss of bleeding protection over time remains to be understood. The authors cite enhanced acid secretion or reduced release of protective prostaglandins after *H pylori* eradication as possible explanations.^5^ Reinfection rates with *H pylori*, another rare (1% to 3% in adult age) but possible explanation, were not monitored. Moreover, because of the uncontrolled use of gastroprotective treatment, the results do not allow answers to the clinically important question of whether guideline-recommended use of proton pump inhibitors may have mitigated the excess bleeding risk enough to reduce its relevance or the impetus of *H pylori* screening and eradication.

## Ongoing Study in Patients with Acute MI

Are the results from HEAT enough to strengthen current guidelines? Considering the limitations mentioned, controversy remains regarding the optimal target group and timing of a routine *H pylori* test-and-treat strategy. Focusing on patients with strong indication for potent antiplatelet therapy but also a higher risk of UGIB may optimize the risk–benefit ratio of the routine *H pylori* screening approach. Whereas HEAT addressed the usefulness of *H pylori* screening in unselected, chronic aspirin users, a study is ongoing to determine the benefit of systematic screening for *H pylori* with subsequent eradication in dual antiplatelet therapy–treated patients with myocardial infarction (HELP-MI [*Helicobacter pylori* Screening to Prevent Gastrointestinal Bleeding in MI Patients]; URL: https://www.clinicaltrials.gov; Unique identifier: NCT05024864). HELP-MI is a cluster-randomized, crossover, clinical trial embedded in a national coronary care registry and conducted at 35 hospitals across Sweden aiming to enroll 20 000 patients with MI during 2 years of recruitment. The primary end point is hospital-treated UGIB during a median follow-up of 2 years. Secondary end points include cardiovascular death; rehospitalization with MI, heart failure, or stroke; and all-cause mortality. All end points will be leveraged from mandatory national registries. Primary results are expected in 2025.

## Summary

The encouraging but inconclusive results from HEAT in unselected low-dose aspirin users underline the need for determining the usefulness of routine *H pylori* screening, as well as optimal timing of *H pylori* eradication, in patients with high-risk coronary artery disease on more potent antithrombotic medication for optimized cardiovascular prevention.

**Figure.**
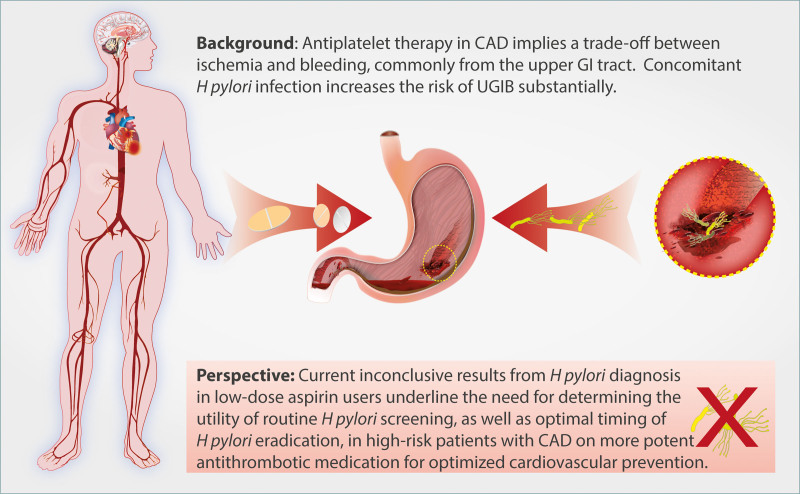
**Time for Helicobacter pylori screening in coronary artery disease?** CAD indicates coronary artery disease; GI, gastrointestinal; and UGIB, upper gastrointestinal bleeding.

## Article Information

### Sources of Funding

Dr Hofmann was supported by the Swedish Research Council (grant 2019-00414), Swedish Heart-Lung Foundation (grant HLF 2021-0273) and the Region Stockholm (clinical research appointment, grant FoUI-974540).

### Disclosures

None.

## References

[1] Han Y Liao Z Li Y Zhao X Ma S Bao D Qiu M Deng J Wang J Qu P. Magnetically controlled capsule endoscopy for assessment of antiplatelet therapy-induced gastrointestinal injury. J Am Coll Cardiol. 2022;79:116–128. doi: 10.1016/j.jacc.2021.10.028 34752902 10.1016/j.jacc.2021.10.028

[2] Sarajlic P Simonsson M Jernberg T Back M Hofmann R. Incidence, associated outcomes, and predictors of upper gastrointestinal bleeding following acute myocardial infarction: a SWEDEHEART-based nationwide cohort study. Eur Heart J Cardiovasc Pharmacother. 2022;8:483–491. doi: 10.1093/ehjcvp/pvab059 34423350 10.1093/ehjcvp/pvab059 PMC9366628

[3] Budzynski J Kozinski M Klopocka M Kubica JM Kubica J. Clinical significance of Helicobacter pylori infection in patients with acute coronary syndromes: an overview of current evidence. Clin Res Cardiol. 2014;103:855–886. doi: 10.1007/s00392-014-0720-4 24817551 10.1007/s00392-014-0720-4

[4] Hellstrom PM Benno P Malfertheiner P. Gastrointestinal bleeding in patients with Helicobacter pylori and dual platelet inhibition after myocardial infarction. Lancet Gastroenterol Hepatol. 2021;6:684–685. doi: 10.1016/S2468-1253(21)00192-8 34391514 10.1016/S2468-1253(21)00192-8

[5] Hawkey C Avery A Coupland CAC Crooks C Dumbleton J Hobbs FDR Kendrick D Moore M Morris C Rubin G; HEAT Trialists. *Helicobacter pylori* eradication for primary prevention of peptic ulcer bleeding in older patients prescribed aspirin in primary care (HEAT): a randomised, double-blind, placebo-controlled trial. Lancet. 2022;400:1597–1606. doi: 10.1016/S0140-6736(22)01843-8 36335970 10.1016/S0140-6736(22)01843-8

